# Canadian Pediatric Intensive Care Adaptations for Critically Ill Adults During the COVID-19 Pandemic: Survey Study

**DOI:** 10.2196/43602

**Published:** 2023-02-10

**Authors:** Evan Parchomchuk, Tanya Holt, Gregory Hansen

**Affiliations:** 1 College of Medicine University of Saskatchewan Saskatoon, SK Canada; 2 Jim Pattison Children's Hospital Saskatoon, SK Canada

**Keywords:** Canada, COVID-19 pandemic, delivery of health care, pediatrics, population health, health care, intensive care, patient care

## Abstract

**Background:**

The COVID-19 pandemic overwhelmed Canadian hospitals with adult admissions. A large number of adult patients required critical care therapies, placing significant strain on hospital resources. In order to decompress adult intensive care units, pediatric intensive care units (PICUs) introduced adapted models of traditional care to lessen these burdens.

**Objective:**

We aimed to evaluate how PICUs across Canada adapted care for the high volumes of critically ill adults.

**Methods:**

A survey containing 40 questions was sent to the medical directors of 14 Canadian PICUs where English was the primary clinical language. The survey was designed to gain perspective on the various adaptations that PICUs instituted during the COVID-19 pandemic.

**Results:**

Of the 13 PICUs that returned survey responses (response rate: 13/14, 93%), 10 (77%) participated in at least one adaptation to support the influx of admitted adults with COVID-19. The key challenges included disorganization, loss of autonomy, and compromised patient care. The significant advantages of these adaptations included a sense of learning and comradery.

**Conclusions:**

Our study highlighted an unpreparedness in critical care surge capacity. During the COVID-19 pandemic, adaptations rapidly emerged in Canada that involved PICUs with adult care. In the future, preplanned adaptations for optimizing robust critical care services should be developed based on what has been learned from the COVID-19 pandemic.

## Introduction

The COVID-19 pandemic dramatically impacted hospital utilization. Many admitted adult patients had prolonged hospital stays, with a large number requiring support in intensive care units (ICUs) [1]. Unfortunately, the number of adult patients who needed critical care soon outpaced the availability of conventional critical care resources [2-4], while the number of children generally did not [5]. As such, 3 variations to traditional pediatric ICU (PICU) models of care became prevalent to treat this influx.

One adaptation was to have pediatric intensivists mange adult patients in the PICU [6]. As pediatric critical care capacity was protected [7], care was standardized, collaboration with adult physicians occurred, licensing was addressed, and team preparedness was ensured [8], mortality outcomes were very favorable [9-12]. A second adaptation was to redeploy PICU staff to adult ICUs. At least two centers [13,14] documented PICU physician, nurse, and physician assistant redeployment after rapid training sessions. Strain between PICU and ICU staff was noted [14], but this diminished as familiarity with the style of practice was established [14]. The final adaption was to have the PICU to act as a unit for ICU overflow that was staffed by adult physicians. As a single pool of critical care resources was distributed to both adult and pediatric patients, equitable considerations became necessary [15-19].

The purpose of this study was to evaluate how PICUs across Canada adapted care for the high volumes of critically ill adults during the COVID-19 pandemic.

## Methods

### Survey Development

PubMed and Google Scholar were searched for articles containing the keywords *COVID-19*, *critical care*, *adults*, and *pediatric intensive care units*. Literature was limited to articles published in the last 2 years, due to the novelty of the COVID-19 pandemic. The literature was retrieved, and the references were reviewed. Following this, 2 pediatric intensive care specialists reviewed the literature and began to create the survey. The survey addressed the following three main adaptations to traditional models of care: opening PICU beds for adult ICU staff and patients, deploying PICU staff to adult ICUs, and managing adult patients with PICU staff in the PICU. Pertinent questions related to these adaptations were reviewed by an independent intensivist to ensure the validity and relevancy of questions. The survey was created and then converted to a REDCap (Research Electronic Data Capture; Vanderbilt University) survey. REDCap was designed to support data capture for research studies, ensure secure web authentication and secure layer encryption, and allow for anonymous participant responses [20]. REDCap was maintained by the University of Saskatchewan.

### Ethics Approval

The survey was approved by the human research ethics board of the University of Saskatchewan (#3248).

### Survey Format

The survey (Multimedia Appendix 1) consisted of 40 possible questions that were divided into 3 domains that corresponded to an adaptation. The three adaptations were titled *opening PICU beds for adult staff and patients*, *deploying PICU staff to adult ICUs*, and *managing adult patients with PICU staff in the PICU*. Each domain had required responses, and subsequent questions would only be displayed, via an embedded branching logic algorithm, for certain responses. If participants did not participate in any of the three adaptations mentioned in the survey, they were immediately directed to the conclusion of the survey.

### Survey Administration

The contact information of 14 Canada medical-surgical PICUs or mixed medical-surgical–cardiac PICUs, where English was the primary clinical language, was gathered from university directories and local sources. In April 2022, a cover letter that introduced the survey and briefly described the content was distributed to the medical directors of each of these PICUs. The link to the REDCap survey was attached to a cover letter that stated “completion and return of the survey implies consent to participate.” Survey answers could be changed prior to submission. Further, 3 reminder emails were sent to the participants every 2 weeks. Coded usernames were stored with responses and later deleted to prevent duplicate entries. No incentive was given for the completion of the survey.

### Data Management and Statistics

Data were collected and managed by using REDCap software. Participant data were anonymous and were analyzed by using IBM SPSS Statistics 28 (IBM Corp). Responses were reviewed for completeness. Proportions were calculated for questions, when applicable. Further, 2 authors (GH and TH) inductively coded all comments as a whole and deductively coded comments from each section into a framework of lessons learned and positive or negative aspects. Representative quotations were agreed upon by all authors.

## Results

### Overview of Survey Responses

A total of 13 survey responses (response rate: 13/14, 93%) were returned by PICU medical directors. All returned surveys were fully completed. Of the 13 PICUs, 3 (23%) had pre-existing pandemic plans that involved adult ICU and PICU collaborations. During the COVID-19 pandemic, 10 (77%) PICUs participated in adult intensive care by opening PICU beds for adult ICU staff and patients (3/13, 23%), deploying PICU staff to adult ICUs (5/13, 38%), and managing adult patients with PICU staff in the PICU (8/13, 62%).

### Opening PICU Beds for Adult Staff and Patients (n=3)

This adaptation was coordinated at the institutional (n=1), regional health authority (n=1), and provincial health authority (n=1) levels. The trigger for the initiation of this model of care was adult ICU capacity being overwhelmed (n=3). The PICUs accepted COVID-19–positive patients (n=1), COVID-19–negative patients (n=1), or both COVID-19–positive patients and COVID-19–negative patients (n=1). A range of 4 to >100 adults were admitted, through this adaptation, over a period of <2 weeks to 10 months. Center responses are summarized in Textbox 1.

**Pediatric intensive care unit (PICU) medical directors’ perspectives on opening PICU beds for adult patients and staff.**

**Negative effects on PICU patient care (n=2)**

1. “Communication and practice style between adult intensivists, registered nurses (RN) and respiratory therapists (RRT) were very different, and so there were difficulties felt on the part of our staff (PICU, RNs) not wanting to pick up shifts because they did not what not deal with adults and adult style of medicine.”
2. “Short staffing in the PICU was amplified, and staff felt they had to sedate their patients more in order to take care of all of the patients.”

**Positive observations for this adaptation (n=3)**

1. “Sense of help during crisis…adult side incredibly grateful.”
2. “Saw more early patient mobilization.”
3. “Good learning experience during periods of surges.”

**Lessons learned (n=3)**

1. “The great work that we do at end-of-life with children becomes evident when you see how that type of care is provided to dying adults.”
2. “A ton of work and planning sent into this – probably easier to replicate in the future.”

### Deploying PICU Staff to Adult ICUs (n=5)

A total of 5 centers deployed registered nurses, 4 deployed registered respiratory therapists, and 1 deployed physicians. No centers deployed social workers, pharmacists, or dieticians. Further, 2 centers coordinated this adaptation at the institutional level, while the others involved all levels, including the regional health authority, provincial health authority, and provincial Ministry of Health levels. The duration of this adaptation ranged from 6 weeks to 8 months, and this adaptation was specifically triggered by projections of ICU admissions, actual ICU admissions, and ICU staff shortages. Center responses are summarized in Textbox 2.

**Pediatric intensive care unit (PICU) medical directors’ perspectives on deploying PICU staff to adult intensive care units.**

**Concerns of ensuring clinical preparedness (n=3)**

1. “Nurses were supposed to be given some training…some got more than others.”
2. “There was minimal preparation provided to the RNs and RRTs. They were initially in more supervised roles on the adult units before taking on patients independently.”
3. “Buddy days for some people, but the majority were RNs with previous adult experience who just went straight to patient care.”

**Negative effects in PICUs with this adaptation (n=2)**

1. “We had to cancel elective surgeries due to lack of RNs in PICU during high volume days.”
2. “The impact was felt more on the RRT side. We often just worked short. The facility did float ward nurses to us more frequently to backfill PICU nursing shortages, but this couldn’t be done with RRTs.”

**Positive observations for this adaptation (n=4)**

1. “Team members returned with some added perspectives they were able to share with all of us on the practice of critical care, during the pandemic, and in general.”

**Lessons learned (n=3)**

1. “There is more in common between PICU and adult ICU than there is different.”
2. “Be transparent with your team. Consider seeking for volunteers before assuming mandatory deployment.”

### Managing Adult Patients With PICU Staff in the PICU (n=8)

The coordination of this adaptation was largely done (n=6) via collaborations among the regional health authority, provincial health authority, and provincial Ministry of Health levels. The volume of adult admissions to Canadian PICUs ranged from 6 to 100 patients during an admission period ranging from 2 weeks to 8 months. Further, 4 centers restricted their admissions to COVID-19–positive patients, 1 restricted its admissions to COVID-19–negative patients, and 3 admitted both COVID-19–positive patients and COVID-19–negative patients. Additionally, 3 centers also limited the adults they cared for to those aged a maximum of 50 years. A total of 4 centers had a pre-existing specialized pediatric transport team, but only 1 center became involved with the interfacility transport of adult patients. To ensure that pediatric capacity for critically ill patients was not compromised, most centers (7/8, 87.5%) used a refined daily approach to managing bed availability in the adult ICUs. This adaptation was triggered by projected and actual surges in hospitalizations, with adult capacity ranging between 100% and 125%.

Center responses are summarized in Textbox 3.

**Pediatric intensive care unit (PICU) medical directors’ perspectives on managing adult patients with PICU staff in the PICU.**

**Measures to ensure that adult care was not compromised (n=8)**

1. “Supervised by adult intensivists.”
2. “Rapid development of policies and procedures, education sessions, shared folders with resource documents”
3. “We arranged for adult subspecialists to consult with us rather than their pediatric versions.”
4. “Adult code simulations”

**Negative effects on PICU patient care (n=3)**

1. “We worked more hours in the month compromising our lifestyle.”
2. “Emotional strain of managing COVID-19 and death of adult patients was different”

**Positive observations for this adaptation (n=7)**

1. “Some exposure to new ways of doing things.”
2. “Adult intensivists understood that were intensivists also.”
3. “Multiple opportunities for collaboration and engagement occurred with our adult ICU colleagues.”

**Lessons learned (n=7)**

1. “Different staffing models explored.”
2. “Surge capacity can be organized and resiliency in moments of national emergency were very developed”
3. “With good back up, adults can be cared for well in a PICU (some patients' families did not want to go back when the time came!).”

### Lack of Engagement and Autonomy (n=8)

The primary theme that arose from PICU directors’ responses was their perceived lack of engagement and autonomy (n=8). They commented that adaptation decisions were “imposed,” they had “no choice,” there was not a venue “for [pushback],” and adaptation decisions were conducted with “excessive zeal.” In one instance, directors “had other models proposed…not accepted.” Moreover, similar reflections were noted after the crisis had subsided, as follows:

Not enough engagement, and not enough push back and not enough power to bring the staff back when the situation improved.

Even with very low (adult ICU admission) numbers, the PICU was not given back to us.

We did not have enough power (to return to regular model of care) when the situation improved.

## Discussion

Our Canadian survey demonstrated a lack of pre-existing COVID-19 pandemic plans among adult ICUs and PICUs. Grappling to find solutions for adult ICU surges, many PICU directors felt uninvolved with the new care modeling and felt that they had compromised care in the process. However, each center described positive learning experiences, which may be useful for future pandemic planning.

The lack of PICU preparedness for adapting to adult ICU surges resulted in concerns from directors. Many were unhappy with their lack of engagement and autonomy in decision-making, leading to adaptations that were not desirable and precipitously instituted. Although the survey did not specifically ask for details of engagement, acute adult surges may have resulted in efficiency and speed being prioritized. Furthermore, several adaptations were initiated by novel committees from provincial pandemic organizations, which may have lacked adequate pediatric representation.

Regardless of the adaptation, many PICU directors suggested that pediatric care was compromised. The adaptations resulted in strategically placed PICU resources being moved, staff shortages, the cancellation of pediatric surgeries, and increased patient sedation due to busier patient assignments. Although the survey was not designed to quantitatively assess these compromises, the sentiments conveyed an environment of suboptimal care in which both pediatric and adult patient morbidities could have occurred.

Despite the adaptations’ negative consequences, some unexpected positive lessons emerged. A sense of esprit de corps and pride emerged, as pediatric intensivists understood the necessity of the adaptions and exhibited a willingness to assist with the adult ICU surge. Positive relationships between adult and pediatric intensivists were fostered, as there was a recognition of the commonalties between adult and pediatric care. Clinically, exposure to adult care and protocols provided unique perspectives and learning opportunities. Most importantly, some centers felt empowered when reflecting on what was accomplished.

Going forward, the concerns that arose from Canadian PICU directors during the COVID-19 pandemic suggest a necessary preparedness for future pandemics. Provincial commitments are required to ensure dedicated funding for existing adult and pediatric critical care beds, the continuation of opening additional beds, and the recruitment and training of critical care physicians. New models of critical care could be considered that view critical care as a service line and have adaptable transition points between PICUs and adult ICUs. Intraprovincial collaborations that involve institutions, medical leadership, and health authorities at the local, regional, and provincial levels are needed to address pediatric and adult surge planning with potential adaptations that involve cross coverage. Lastly, interprovincial collaborations could be initiated, as many provinces had to rely on cross-border patient transports. However, PICU directors were very clear that any potential shifts must be guided by transparency and multidisciplinary team engagement.

The survey had several potential limitations. Although our total response rate was 93% (13/14), there is a risk of sampling bias with only surveying medical directors from English-speaking PICUs. Furthermore, despite our best efforts to create a survey that avoided leading questions, our data may reflect response biases due to the nature of the pandemic and its progression. As this study addressed large challenges for critical resources, voluntary response bias may have resulted in the overrepresentation of PICU medical directors with strong opinions. Finally, whether the findings here can be generalized to PICUs outside Canada cannot be determined.

Across Canada, the abrupt need for critical care surge capacity resulted in adaptations of PICU care. Although several negative aspects of these adaptations were revealed, many lessons were learned, and some positive feedback emerged. These considerations may be important for ensuring that robust critical care services can be rapidly and efficiently mobilized during future pandemics.

## Appendix

Multimedia Appendix 1 Survey questionnaire.

## Abbreviations

ICU: intensive care unit
PICU: pediatric intensive care unit
REDCap: Research Electronic Data Capture
