# Supplementary material for: Canadian Pediatric Intensive Care Adaptations for Critically Ill Adults During the COVID-19 Pandemic: Survey Study
Source: JMIR Pediatr Parent. 2023 Feb 10;6:e43602. doi: 10.2196/43602 (PMC9960024; doi:10.2196/43602)
Supplement: Multimedia Appendix 1 [file pediatrics_v6i1e43602_app1.docx]

**Survey Questionnaire**

1. Prior to the COVID-19 pandemic, did your centre have a pre-existing pandemic plan that included adult and pediatric ICUs supporting one another if required?

Yes No

2. At any time during the pandemic, did your PICU participate in the care of adult intensive care patients by a) opening PICU beds for adult ICU staff and patients; b) deploying PICU staff to adult ICUs; and/or c) managing adult patients with PICU staff in the PICU?

Yes No

If Yes, proceed to question 3.

If No, proceed to the conclusion.

**Opening PICU Beds for Adult ICU Staff and Patients**

3. Did you open PICU beds for adult patients to be managed by adult ICU staff?

Yes No

If Yes, proceed to question 4

If No, proceed to question 14

4. How was this initially coordinated? i.e. Was it done at the level of your institution, regional health authority, provincial health authority and/or provincial Ministry of Health?

5. Did you feel like your PICU had adequate engagement and autonomy in the decision making to adopt this model of adult care?

6. How did you ensure that pediatric capacity for critically ill patients was not compromised as PICU beds were reallocated to adults?

7. During the COVID-19 pandemic, what was the specific trigger to initiate this model of care?

8. Approximately how many adult patients were admitted to your PICU under this model of care?

9. What type of adult patients were admitted?

COVID+ only

Non COVID patients

Both COVID and nonCOVID+ patients

10. For how long did your adopt this model of care?

Provide Range

11. Do you feel that this model of care had any negative impacts on PICU patient care?

12. Were there any unforeseen positives that your PICU encountered with this model of care?

13. Were there any lessons learned by your PICU from this model of care?

**Deploying PICU Staff to Adult ICUs**

14. Did you deploy PICU staff to adult ICUs?

Yes No

If Yes, proceed to question 15

If No, proceed to question 25

15. Did you deploy:

RNs

RTs

MDs

Social workers

Pharmacist

Dietitians

16. How was this initially coordinated? i.e. Was it done at the level of your institution, regional health authority, provincial health authority and/or provincial Ministry of Health?

17. Did you feel like your PICU had adequate engagement and autonomy in the decision making to adopt the deployment of pediatric expertise?

18. How did you ensure that pediatric capacity for critically ill patients was not compromised by the deployment of pediatric expertise?

19. During the COVID-19 pandemic, what was the specific trigger to initiate this model of care?

20. How did the team ensure clinical preparedness for safe deployment?

21. For how long where pediatric clinicians deployed to adult ICUs?

Provide date range

22. Do you feel that the deployment had any negative impacts on PICU patient care?

23. Were there any unforeseen positives that your PICU encountered with the deployment?

24. Were there any lessons learned by your PICU from the deployment?

**Managing Adult patients with PICU staff in the PICU**

25. Did you mange adult patient with PICU staff in the PICU?

Yes No

If Yes, proceed to question 26

If No, proceed to conclusion

26. How was this initially coordinated? i.e. Was it done at the level of your institution, regional health authority, provincial health authority and/or provincial Ministry of Health?

27. Did you feel like your PICU had adequate engagement and autonomy in the decision making to adopt this model of adult care?

28. How did you ensure that pediatric capacity for critically ill patients was not compromised as PICU beds were reallocated to adults?

29. During the COVID-19 pandemic, what was the specific trigger to initiate this model of care?

30. How did your ensure adult patient care was not compromised and standard of care was provided while the PICU team expanded their scope of practice?

31. Approximately how many adult patients were admitted to your PICU under this model of care?

32. What type of adult patients were admitted?

COVID+ only

Non COVID patients

Both COVID and nonCOVID+ patients

33. Did you restrict the age of adults you cared for?

Yes No

34. If #33 answered “Yes”, then

Provide age restriction

35. Does your pediatric ICU have a dedicated transport team?

Yes No

36. If #35 answered “Yes”, then Did you pediatric transport team transport adult patients?

Yes No

37. For how long did your adopt this model of care?

Provide date range

38. Do you feel that this model of care had any negative impacts on PICU patient care?

38. Were there any unforeseen positives that your PICU encountered with this model of care?

40. Were there any lessons learned by your PICU from this model of care?

**Conclusion**

Thank you for you participation!
